# Bicaudal-C Post-transcriptional regulator of cell fates and functions

**DOI:** 10.3389/fcell.2022.981696

**Published:** 2022-09-07

**Authors:** Megan E. Dowdle, Charlotte R. Kanzler, Cole R. K. Harder, Samuel Moffet, Maya N. Walker, Michael D. Sheets

**Affiliations:** Department of Biomolecular Chemistry, School of Medicine and Public Health, University of Wisconsin, Madison, WI, United States

**Keywords:** mRNA translation, embryonic cell fate, Bicaudal-C family RNA binding protein 1 (Bicc1), post-transcripional control, cell fate and differentiation

## Abstract

Bicaudal-C (Bicc1) is an evolutionarily conserved RNA binding protein that functions in a regulatory capacity in a variety of contexts. It was originally identified as a genetic locus in *Drosophila* that when disrupted resulted in radical changes in early development. In the most extreme phenotypes embryos carrying mutations developed with mirror image duplications of posterior structures and it was this striking phenotype that was responsible for the name Bicaudal. These seminal studies established Bicc1 as an important regulator of *Drosophila* development. What was not anticipated from the early work, but was revealed subsequently in many different organisms was the broad fundamental impact that Bicc1 proteins have on developmental biology; from regulating cell fates in vertebrate embryos to defects associated with several human disease states. In the following review we present a perspective of Bicc1 focusing primarily on the molecular aspects of its RNA metabolism functions in vertebrate embryos.

## Introduction

The advent of omics technologies has led to the identification of many new and novel RNA binding proteins (RBPs). Strategies such as RNA interaction capture have identified hundreds of RBPs, many of which were previously not known to associate with RNAs or be involved in RNA metabolism (Castello et al., 2016). Many RBPs are post-transcriptional regulators of gene expression that function by participating in one or more steps of mRNA metabolism; from splicing and export out of the nucleus, to subcellular localization, translation and degradation in the cytoplasm (Perez-Perri et al., 2018, 2021). Precedents from well-studied examples suggest that each protein binds to hundreds of RNAs to form a regulatory network (Hentze et al., 2018; Gebauer et al., 2021). Each individual network forms a functional unit of regulation used by cells to adapt to changing conditions or modify cellular functions as part of a developmental program.

In contrast to their identification and molecular characterization *en masse*, the contribution and relevance of individual RBPs to specific aspects of biology has advanced much more slowly. The biological importance of individual RBPs for specific biological processes has often been revealed through either deregulation *via* increases or decreases in expression or structural changes as a result of mutation. The challenge is to define the underlying molecular mechanisms and the networks organized around a particular RBP and functionally connect these to the relevant biology.

Bicaudal-C (Bicc1) is a highly conserved RNA binding protein that functions in a regulatory capacity in many different contexts. It was originally identified as a genetic locus in *Drosophila* that when disrupted, resulted in dramatic changes in early development: embryos carrying mutations developed with mirror image duplications of the posterior structures (Bull, 1966; Mohler and Wieschaus, 1986). These initial studies established Bicaudal-C as an important regulator of *Drosophila* development. Motivated by these findings, developmental biologists identified Bicc1 orthologs and applied a number of strategies to functionally connect this protein to specific embryonic processes. What was revealed was the broad fundamental impact that Bicc1 proteins have on developmental biology and regulating cell fates in vertebrate embryos. In the following review we present a perspective of Bicc1, focusing primarily on the molecular aspects of its RNA metabolism functions in vertebrate embryos.

### Bicaudal-C maternal regulatory protein discovered in *Drosophila*

One of the most critical issues in oogenesis and embryogenesis is defining how the egg and embryo establish polarity to initiate body patterning. An early study published in 1966 on this topic focused on the genetic causes of the double-abdomen or “bicaudal” phenotype in *Drosophila* embryos (Bull, 1966). Genetic analysis revealed that the bicaudal phenotype arose from maternal mutations that could be assigned to specific chromosomal regions, though not at high resolution (Bull, 1966). Subsequent studies identified the gene responsible and named it Bicaudal-C (BicC, the fly ortholog of vertebrate Bicc1) (Mohler and Wieschaus, 1986; Mahone et al., 1995). The predicted Bicaudal-C protein is composed of several putative RNA binding domains and biochemical experiments demonstrated that the protein could efficiently bind to Poly (U) *in vitro* (Mahone et al., 1995). The first evidence that Bicaudal-C functions as a translational repressor came from observations that *Drosophila* BicC loss-of-function mutants affect anterior-posterior patterning as a result of ectopic and premature translation of the posterior determinant oskar (Saffman et al., 1998). Subsequent analysis revealed that Bicaudal-C was conserved throughout the animal kingdom and the first vertebrate ortholog was identified and characterized from *Xenopus* (Wessely and De Robertis, 2000), followed shortly by a mammalian ortholog (Wessely et al., 2001).

**FIGURE 1:**
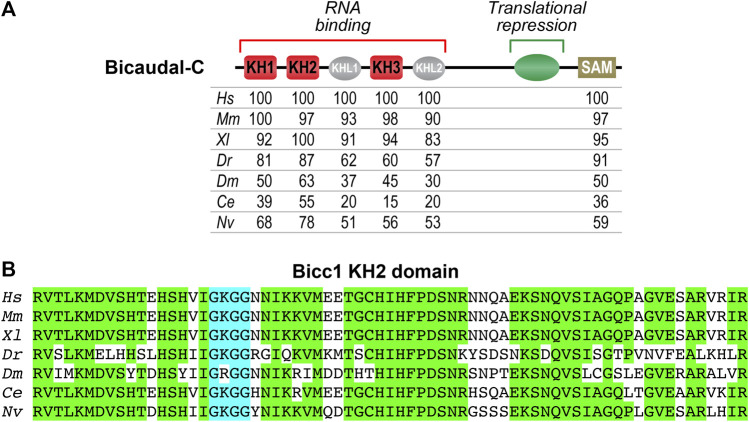
Evolutionary conservation of Bicaudal-C proteins **(A)** Diagram of the Bicaudal-C protein highlighting key features. The N-terminal region contains three KH domains (KH1, KH2 and KH3) involved in RNA recognition and binding. The C-terminal region contains a SAM domain that mediates protein-protein interactions and promotes the formation of polymers. The central region of the protein (aa505-806 of *Xl*Bicc1) was defined through the use of the MS2 tethered function assays as being sufficient for translational repression (Zhang et al., 2013). Below the diagram are amino acid identities comparing Bicc1 proteins from different species (*Hs*–*Homo sapiens*, *Mm*–*Mus musculus*, *Xl*–*Xenopus laevis*, *Dr*–*Danio rerio*, *Dm*–*Drosophila melanogaster*, Ce–*Caenorhabditis elegans, Nv*–*Nematostella vectensis*). The numbers indicate the percent amino acid identity compared to *Hs* Bicc1. The different domains are those identified via the NCBI database. **(B)** Amino acid sequence comparison of the highly conserved KH2 domains from different species. This domain is a key structural feature for recognition of specific mRNAs by Bicc1 proteins and one of the most conserved features of these proteins. Amino acids that are identical are highlighted in green. The KH2 domain’s GXXG motif (GGKG) is highlighted in blue.

Comparison of Bicc1 proteins from different organisms reveals several conserved features (Figure 1). The amino terminal half of the protein contains three hnRNP K homology (KH) domains (KH1, KH2 and KH3), with the KH3 domain being flanked on each side by KH-like (KHL) domains. KH domains are found in a wide variety of nucleic acid-binding proteins, especially those that function in RNA recognition (Valverde et al., 2008; Nicastro et al., 2015). Canonical KH domains contain GXXG motifs and KHL domains have the same general architecture but lack GXXG motifs. The carboxyl terminus of Bicc1 proteins contains a SAM (sterile alpha motif) domain that mediates protein-protein interactions (Rothé et al., 2018). Sequence conservation among vertebrates is very high, supporting the idea that functional analysis in model organisms can be related to potential Bicc1-related clinical conditions observed in human patients (Figure 1).

## Bicaudal-C regulated biological processes

### Anterior-posterior patterning

**FIGURE 2:**
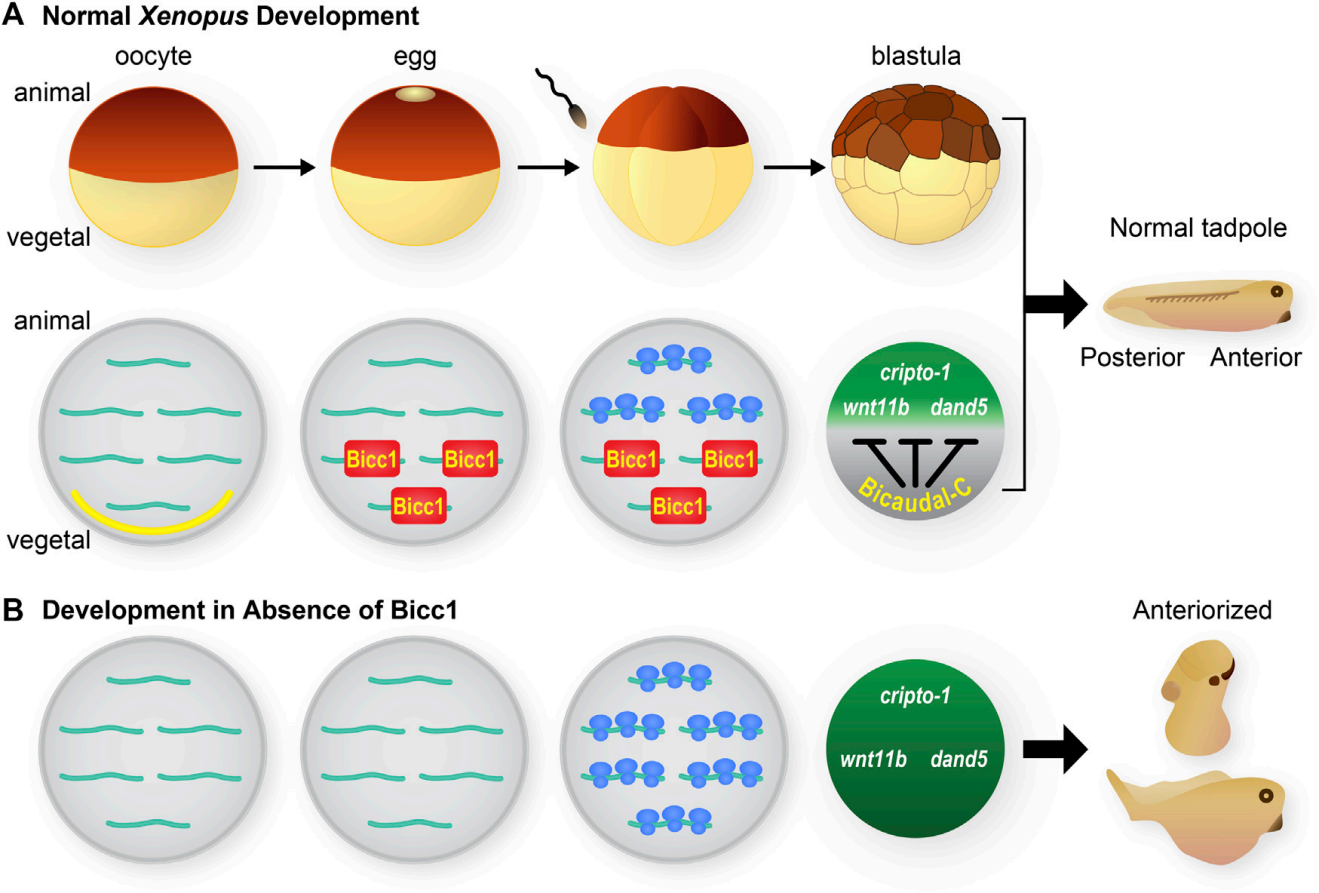
Xenopus Bicaudal-C, regulator of maternal mRNA translation and cell fates **(A)** The diagram at the top depicts the early stages of *Xenopus* development. Oocytes mature into eggs that are competent to be fertilized. Following fertilization the embryos undergoes a series of rapid cleavage divisions to generate blastula stage embryos. The initial stages of embryonic development occur in the absence of zygotic transcription and rely on the regulated use of maternal mRNAs and proteins. During normal development *bicc1* mRNA is localized to the vegetal cortex of fully-grown oocytes (yellow section at vegetal pole) (Wessely and De Robertis, 2000). During oocyte maturation the *bicc1* mRNA is released from the cortex and at the same time becomes translationally active to generate Bicc1 protein (Park et al., 2016). Bicc1 protein is distributed in a vegetal to animal gradient in embryos where it binds to specific mRNAs and represses their translation. Several of these target mRNAs (*cripto-1*, *wnt11b* and *dand5*) encode cell fate regulatory proteins. Bicc1 normally modulates the synthesis of these proteins and their activities contribute to the formation of a tadpole with anterior-posterior polarity. **(B)** Development in the absence of Bicc1. To create embryos that lack Bicc1 oocytes are injected with anti-sense oligonucleotides to specifically degrade the Bicc1 mRNA. Treated oocytes are matured and then converted to embryos using the host transfer technique (Park et al., 2016). Bicc1 target mRNAs are translated inappropriately in embryos depleted of Bicc1 and the encoded proteins accumulate to higher levels. As a consequence Bicc1 depleted embryos develop into tadpoles with an excess of anterior cell types and structures–an anteriorized phenotype (Park et al., 2016).

**FIGURE 3:**
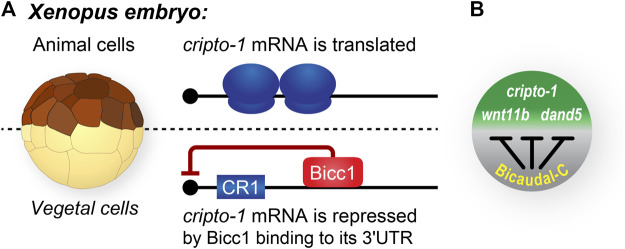
Bicaudal-C spatially regulates the accumulation of cell fate determinants in *Xenopus* embryos. **(A)** *Xenopus* blastula stage embryos are polarized with pigmented animal cells and non-pigmented vegetal cells. In general, mRNAs encoding cell fate determinants, such as *cripto-1* are uniformly distributed throughout the embryo. In contrast, the Bicc1 repressor protein is present in a vegetal to animal gradient in which the highest concentration of protein is present in the vegetal cells and the lowest in the animal cells (Park et al., 2016). **(B)** Bicc1 target mRNAs are translated into protein with different efficiencies due to the their position in the Bicc1 repression gradient (Sheets et al., 2017).

The first vertebrate Bicc1 ortholog was identified in *Xenopus* (Wessely and De Robertis, 2000). The *bicc1* mRNA is highly expressed maternally and localized to vegetal cells of developing embryos (Figure 2A). Subsequent analysis identified Bicc1 as a regulator of maternal mRNA translation and in particular translation of the *cripto1 (tdgf1.3)* mRNA that encodes a cell fate regulatory protein (Figure 3) (Zhang et al., 2013). These connections provided the motivation for analyzing the consequences of removing Bicc1 from vertebrate embryos. To address this issue, *Xenopus* embryos depleted of maternal Bicc1 were generated using the host transfer approach (Park et al., 2016). Bicc1-depleted embryos develop with an expansion of anterior structures (enlarged heads and cement glands) and cell types at the expense of posterior cells (Figure 2B). These morphological changes are accompanied by characteristic changes in the expression of anterior/posterior-specific mRNAs (Park et al., 2016). The anteriorized phenotype due to Bicc1 depletion is the opposite of the phenotypes caused by depleting *Xenopus* embryos of maternal mRNAs encoding either the cripto1, wnt11b or dand5 (coco) cell fate regulatory proteins (Tao et al., 2005; Bates et al., 2013). Since these mRNAs are known Bicc1 targets, these phenotypes suggest that a Bicc1 depletion increases the translation of these targets, a suggestion supported by data demonstrating that Bicc1 represses *cripto1*, *dand5* and *wnt11b* mRNA reporters (Zhang et al., 2013; Park et al., 2016). Thus, maternal Bicc1 in *Xenopus* embryos functions in anterior-posterior patterning by modulating the translation of mRNAs encoding cell fate determinants.

### Left-right patterning

The asymmetric placement of the internal organs is critical for embryonic development of all animal species and is referred to as Left-Right (L-R) patterning (Nakamura and Hamada, 2012; Hamada, 2020). This patterning relies upon an elaborate series of well-orchestrated cellular and molecular processes. Asymmetry is initiated and driven by rotating cilia in cells of the left-right organizer (LRO) which generate an asymmetric flow. This flow is relayed to the lateral plate mesoderm (LPM) to differentially activate the Nodal signaling pathway. While the molecular cues that establish and regulate the embryonic L-R axis have been known for several years, Bicc1’s role emerged relatively recently. BICC1 mutations in mouse or morpholino treatments in *Xenopus* or zebrafish embryos both revealed that disrupting Bicc1 expression leads to heterotaxy and defects in L-R patterning (Maisonneuve et al., 2009). Heterotaxy is the abnormal arrangement and structure of the abdominal organs, especially the heart. Recent evidence suggests that L-R defects due to Bicc1 are at least in part due to disrupted Bicc1 regulation of the mRNA encoding the Nodal inhibitor dand5 (Maerker et al., 2021; Minegishi et al., 2021). The results from these studies demonstrated the central role of Bicc1 RNA regulation in controlling L-R patterning.

### Kidney function

Multiple lines of evidence have demonstrated the importance of Bicc1 for maintaining normal kidney functions. Targeted mutations of mouse *Bicc1* as well as morpholino disruption of *Xenopus* and zebrafish Bicc1 orthologs in developing embryos all result in abnormal kidneys that closely resemble defects due to polycystic kidney disease (PKD) (Cogswell et al., 2003; Tran et al., 2007, 2010; Maisonneuve et al., 2009; Bouvrette et al., 2010). In *Drosophila*. Renal function is carried out by the Malpighian tubules and the nephrocytes. *Drosophila* BicC mutants develop with malformed Malpighian tubules that resemble the renal cysts found in vertebrates in which BICC1 expression is disrupted (Gamberi et al., 2017). The observed defects were attributed in part to disrupting the BicC regulation of the *myc* mRNA. While there is abundant evidence demonstrating the importance of Bicc1 in kidney functions, defining the underlying molecular mechanisms involved is an active area of investigation.

## Bicaudal-C target mRNAs

### *Xenopus cripto1* mRNA and the spatial control of translation

The Nodal signaling pathway is critical for vertebrate development and Cripto proteins are key components of this signaling (Tao et al., 2005; Schier, 2009; Nagaoka et al., 2012; Klauzinska et al., 2014). The *Xenopus cripto1* mRNA is maternally expressed and its translation is both temporally and spatially regulated (Zhang et al., 2009). Specifically, the entire *cripto1* mRNA population in oocytes and eggs is translationally repressed. Then, after fertilization, the fraction of *cripto1* mRNA present in vegetal cells remains repressed while the mRNA in animal cells is actively translated (Figure 2A). Lineage-specific injection of reporter mRNAs containing the 3′UTR from the *cripto1* mRNA recapitulated the spatially regulated translation observed in embryos (Zhang et al., 2009). These results demonstrated that sequences in the 3′UTR of the *cripto1* mRNA were sufficient to direct embryonic translational repression and suggested that vegetal cells contain a repressor that functions via these 3′UTR sequences.

Multiple pieces of evidence identified Bicc1 as the vegetal cell-specific translational repressor of *cripto1* mRNA (Figures 2, 3) (Zhang et al., 2009, 2013). First, *Xenopus bicc1* mRNA and protein are restricted to vegetal cells of developing embryos, suggesting that the *cripto1* mRNA is not repressed in animal cells because these cells lack the Bicc1 repressor (Figures 2, 3) (Wessely and De Robertis, 2000; Park et al., 2016). This idea was tested with reporter mRNAs that were efficiently repressed in animal cells only when the animal cells ectopically expressed Bicc1 (Zhang et al., 2013). Second, the endogenous *cripto1* mRNA as well as the *cripto1* reporter mRNA were both efficiently bound by Bicc1 *in vivo* (Zhang et al., 2013). Third, a 32-nucleotide Bicc1-binding site was identified within the *cripto1* mRNA 3′UTR using biochemical approaches and reporter assays (Zhang et al., 2013, 2014; Dowdle et al., 2017). Together, these results demonstrate that *Xenopus* Bicc1 is a vegetal cell-specific repressor responsible for spatially regulating *cripto1* mRNA translation in maternal stage embryos.

### *Xenopus* Bicc1 forms a translational repression gradient in developing embryos

The *Xenopus* maternal *bicc1* mRNA is first expressed during the early stages of oogenesis and as oogenesis proceeds the mRNA is localized to the vegetal cortex of fully-grown oocytes (Wessely and De Robertis, 2000). Then, during oocyte maturation, the *bicc1* mRNA is released from the cortex and simultaneously translationally activated to produce Bicc1 protein (Figure 2) (Park et al., 2016). These processes create a vegetal to animal (V-A) gradient of Bicc1 protein and its repression activity that is passed on to embryonic cells after fertilization. Bicc1 repression functions are highest in vegetal cells, lowest in animal cells, and the marginal zone cells exhibit intermediate levels of activity (Park et al., 2016). There are multiple Bicc1 target mRNAs, raising the possibility that the Bicc1-repression gradient gives rise to secondary gradients of proteins encoded by Bicc1 targets. Such gradients have the potential to differ based on the affinity of individual target mRNAs for Bicc1 and the efficiency of translational repression (Sheets et al., 2017).

### Bicc1 regulated mRNAs in mouse

The analysis of proteins whose expression increased in Bicc1−/− mouse kidney tubules lead to the identification of the mRNAs encoding the adenylate cyclase-6 (Adcy6) and protein kinase inhibitor (PKIa) proteins as some of the first Bicc1 regulated mRNAs (Piazzon et al., 2012). These mRNAs were associated with Bicc1 in immunoprecipitation experiments and the translation of these mRNAs via their 3′UTRs was inhibited by Bicc1 in reporter assays. Results from co-expression network–analysis of microarray experiments, identified Bicc1 as a genetic determinant of osteoblastogenesis in part through its regulation of Pkd2 transcript levels (Lemaire et al., 2015). Additionally, comparison of mRNA changes in pancreatic cells between WT and Bicc1 knockouts indicated that Pkd2 functions downstream of Bicc1 in preventing pancreatic cyst formation (Mesner et al., 2014). Thus, Pkd2 mediates Bicc1 activity in multiple contexts. Recent studies identified mouse *dand5* as a Bicc1 regulated mRNA and a key component of the L-R patterning pathway (see below) (Minegishi et al., 2021).

### The *Xenopus* Bicc1 network

mRNA binding proteins (RBPs) manifest themselves phenotypically through binding and impacting the function of specific mRNAs. Hence, the key issue for each RBP is identifying the relevant target mRNAs. The first suspected target for BicC was the *Drosophila oskar* mRNA (Mahone, Saffman and Lasko, 1995; Saffman et al., 1998). It was observed that in BicC−/− mutant *Drosophila* embryos *oskar* mRNA was translated at higher levels than in wild-type embryos. A subsequent study in *Drosophila* identified multiple BicC target mRNAs using microarrays that included the mRNA encoding *BicC* itself (Chicoine et al., 2007).

In *Xenopus* embryos 63 Bicc1 target mRNAs were identified using RNA immunoprecipitation sequencing (RIP-SEQ) (Zhang et al., 2013). Many of these targets were validated using follow-up experiments, including luciferase reporter assays in which the reporters contained the 3′UTRs of potential targets. Among the validated targets were mRNAs such as *cripto1* and *dand5* that encode known signaling proteins important for fate decisions in *Xenopus* and other vertebrate embryos (Tao et al., 2005; Bates et al., 2013). Subsequent studies in *Xenopus* also identified the *wnt11b* and *gdf3* mRNAs as additional authentic Bicc1 targets (Park et al., 2016; Maerker et al., 2021). These results defined the Bicc1 target mRNA network and provided a foundation for understanding the molecular events that underlie Bicc1 regulated biological events during vertebrate development and potentially other contexts.

## Mechanisms of mRNA binding and regulation

### mRNA features required for Bicaudal-C binding

**FIGURE 4:**
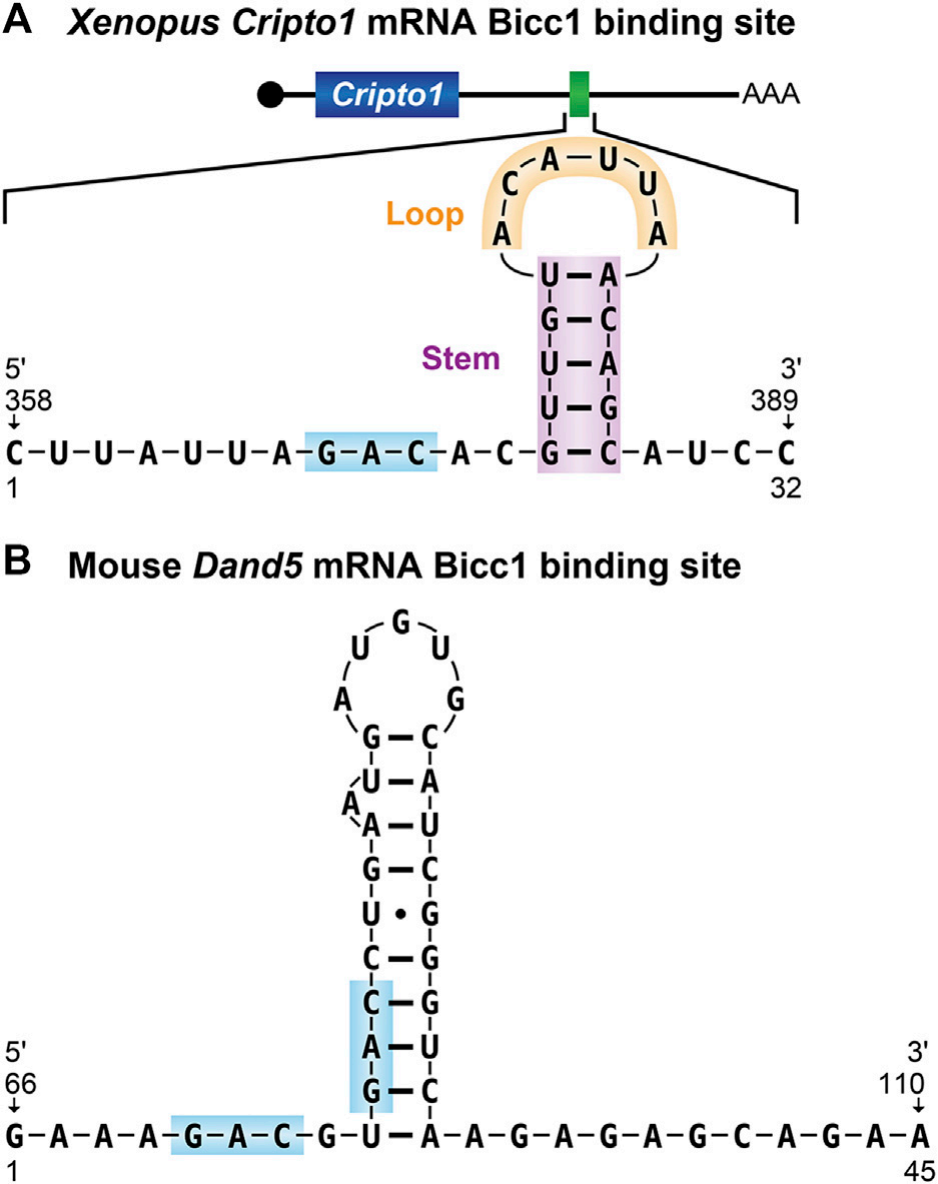
Bicaudal-C RNA binding sites **(A)** The 32-nucleotide Bicc1 binding site from the *Xenopus cripto-1* mRNA. The predicted stem-loop structure identified from the 3′UTR of the *cripto-1* mRNA is shown (Zhang et al., 2013, 2014). **(B)** The Bicc1 binding site from the 3′UTR of the mouse *dand5* mRNA (Minegishi et al., 2021). GAC sequence motifs present in both binding sites are highlighted.

The most well characterized Bicc1-RNA interface is recognition of the *Xenopus cripto1* mRNA, as described above. A combination of EMSA (electrophoretic mobility shift assays), RNAse footprinting, and reporter experiments demonstrated that Bicc1 recognizes the *cripto1* mRNA via a 32-nucleotide region located within its 3′UTR that is predicted to form a stem-loop structure (Figure 4) (Zhang et al., 2013, 2014; Dowdle et al., 2017, 2019). The double-stranded properties of the stem along with the nucleotide sequence of the loop were found to be important determinants of binding (Zhang et al., 2014). However, the stem-loop alone was not sufficient for Bicc1 binding, indicating that other important features remain to be defined (Zhang et al., 2014).

Recent work in *Xenopus*, zebrafish, and mouse identified Bicc1 as an important factor downstream of flow sensing in L-R patterning via regulation of the *dand5* and *gdf3* mRNAs. In *Xenopus* embryos Bicc1 functions in pre-flow regulation in the left-right organizer by stabilizing the *gdf3* and *dand5* mRNAs via their 3′UTRs (Maerker et al., 2021). In post-flow stages, Bicc1 functions as a potent repressor of *dand5* translation mediated by the proximal-most 139 nucleotides of the *dand5* 3′-UTR. Deleting the distal 36 nucleotides of this region renders the *dand5* mRNA insensitive to Bicc1 regulation and as such the region was referred to as the Bicc1 response element. This post-transcriptional mechanism is conserved and in mouse an analogous proximal element of the *dand5* 3′-UTR is required for Bicc1-dependent flow-mediated mRNA decay and translational inhibition (Minegishi et al., 2021). Bicc1 also represses translation of the *Xenopus gdf3* mRNA via its 3′UTR (Maerker et al., 2021). Because the gdf3 protein also influences Nodal signaling it is clear there are multiple paths by which Bicc1 influences L-R patterning.

Studies in mouse found that the first 200 nucleotides of the *dand5* 3′UTR were both necessary and sufficient for Bicc1 regulation and binding (Minegishi et al., 2021). To define sequences with the potential for Bicc1 binding, RNA Bind-n-Seq experiments were performed using lysates generated from FLAG-tagged Bicc1-expressing 293FT cells as the source of protein. The results revealed the enrichment of primarily two sequence motifs YGAC and GACR. These motifs were present in the critical regions of both mouse and *Xenopus dand5* 3′-UTRs, adjacent to one another in a potential tandem bipartite organization. These motifs as well as their organization are conserved in 3′UTRs of *dand5* mRNAs from mammals, *Xenopus* and medaka. It was noted that while other Bicc1 regulated mRNAs, such as the Zebrafish *dand5* mRNA and *Xenopus cripto-1* mRNAs, lack such bipartite motifs, they do contain single GAC sequences in their 3′-UTRs. Thus, the generality of the bipartite GAC features for other Bicc1 target mRNAs remains to be established.

Biochemical EMSA experiments demonstrated that wild-type *dand5* RNA substrates were efficiently bound by Bicc1 and RNAs containing changes to the GAC motifs disrupted binding (Minegishi et al., 2021). Accompanying *in vivo* analysis of a *dand5* reporter mRNA containing nucleotide changes of the GAC motif demonstrated that without the intact motif, Bicc1 binding in mammalian cells was inefficient. Together, these results suggest that Bicc1 binding to the Dand5 3′-UTR is mediated by GAC motifs in a bipartite organization (Figure 4).

### Features of the bicc1 protein required for mRNA recognition

The structural features needed for efficient and specific RNA binding by Bicc1 have been analyzed most extensively with the *Xenopus* protein. Biochemical experiments combined with the *in vivo* results from immunoprecipitation-RT-QPCR analyses demonstrated that the *Xenopus* Bicc1 N-terminal region (amino acid residues 1–505) was necessary and sufficient for specific RNA binding while the C-terminal region lacked activity (Zhang et al., 2013, 2014; Dowdle et al., 2017, 2019).

The N-terminal region of Bicc1 contains three evolutionarily conserved KH domains (KH1, KH2 and KH3) and 2 KH-like (KHL1 and KHL2) (Figure 1). Multi-KH domain containing proteins often use only a specific subset of domains for RNA binding (Nicastro et al., 2015; Farina et al., 2003; Chao et al., 2010). To address this issue with Bicc1, variants of the *Xenopus* protein lacking one or more KH domains were examined for RNA binding both *in vivo* and *in vitro*. All three KH domains are required for specific RNA binding, suggesting a potentially complex mechanism of RNA recognition (Dowdle et al., 2019).

Canonical KH domains contain conserved GXXG motifs that provide contacts with the backbone of RNA substrates (Valverde et al., 2008; Nicastro et al., 2015). In studies of model KH domain-containing proteins, GDDG substitutions abolish the RNA binding functions of individual domains without perturbing overall protein architecture or the function of adjacent domains (Hollingworth et al., 2012). Analyzing a collection of *Xenopus* Bicc1 proteins with GDDG substitutions in individual KH domains revealed that the GXXG motif of the KH2 domain (GKGG) was a major determinant of Bicc1 RNA binding (Dowdle et al., 2019). Consistent with its functional importance, the KH2 domain and its GKGG motif are two of the most highly conserved features of Bicc1 proteins (Figure 1).

Recent work analyzing mouse Bicc1 found a similar primary importance of the KH2 domain and its GKGG motif for recognition of the *dand5* mRNA (Minegishi et al., 2021). In addition, a requirement for the KH1 domain via its GXXG motif for *dand5* mRNA binding was also observed. This latter observation differs from what was observed with *Xenopus* Bicc1 and its binding to the *cripto1* mRNA where KH1 and its GXXG motif were not critical for RNA binding (Dowdle et al., 2019). These findings raise the possibility that Bicc1 recognizes different mRNAs and different sequences through the use of distinct combinations of KH domains.

## mRNA regulation by Bicaudal-C

Identification of the BicC gene in *Drosophila* provided the amino sequence of the predicted protein and revealed the presence of putative KH domains (Saffman et al., 1998). This identification immediately suggested that the KH domains were important for the mRNA binding activities of BicC as similar domains in other proteins were known to mediate their interactions with RNA targets (Valverde et al., 2008; Nicastro et al., 2015). In contrast, the protein sequence did not reveal any clues to suggest how BicC regulates mRNAs and addressing this issue required unbiased experimental analysis.

To begin defining *Xenopus* Bicc1’s mRNA regulatory functions, the protein and different subregions was analyzed using an MS2 tethering assay (Zhang et al., 2013). One of the powerful aspects of this assay is that it allows the RNA regulatory functions to be analyzed independent of Bicc1’s RNA binding activity. The full-length and the C-terminal region of Bicc1 (aa505-805) were highly effective at mRNA repression (Figure 1). In contrast, the N-terminal region and the SAM domain exhibited no detectable repression activities. Bicc1 repression was due to disrupting translation and not due to differences in mRNA stability or the removal of poly(A). These functions are conserved, as the C-terminal region of human Bicc1 also repressed efficiently (Zhang et al., 2013). Thus, the C-terminal region of *Xenopus* Bicc1 is sufficient for translational repression and as such represents the Bicc1 repression domain analogous to what has been defined for the human Pumilio protein (Figure 1) (Enwerem et al., 2021).

One of the principal mechanisms by which Bicc1 represses target mRNAs is through interactions with the major eukaryotic mRNA deadenylase, the Ccr4-Not deadenylase cmplex (CNOT complex). Studies in *Drosophila* ovarian extracts were the first to observe that BicC interacts with the Ccr4 and NOT3/5 subunits of the CNOT complex (Chicoine et al., 2007). The interaction with the NOT3/5 protein was direct as it was also detected with recombinant protein produced in *E. coli*. BicC mutant embryos exhibited a stage-dependent impact on the length of the poly(A) tails of target mRNAs. This lead to a model in which BicC recruits the CNOT complex to these mRNAs and impacts their translation by poly(A) removal (Chicoine et al., 2007).

Mammalian CNOT1 was identified as an interaction partner with mouse Bicc1 in HEK293 cells (Leal-Esteban et al., 2018). Further connections between Bicc1 and the CNOT complex were investigated in the context of left/right patterning and mouse *dand5* mRNA regulation. It was observed that both the CNOT1 and CNOT3 proteins co-immunoprecipitated with mouse Bicc1 and these interactions depend upon the KH domains, but not the SAM domain or the presence of RNA (Minegishi et al., 2021). These results contributed to a model in which Bicc1 in cells of the mouse node engages with the CNOT complex to regulate *dand5* mRNA.

Other evidence suggests that Bicc1 interacts with microRNAs (miRNAs) and their associated protein components to direct mRNA repression (Tran et al., 2010; Piazzon et al., 2012). In contrast to the early work done in *Drosophila*, Bicc1 did not impact the poly(A) tail of *Adcy6* target mRNA in a mouse model of polycystic kidney disease (Piazzon et al., 2012). Instead, it was hypothesized that Bicc1 was required downstream of the pri-miRNA processing enzyme Dicer to mediate an interaction between the 3′UTR of target mRNAs and the Ago2 protein, in a process that required both cognate microRNAs and the Bicc1 SAM domain (Piazzon et al., 2012). Recent studies of left-right patterning in *Xenopus* embryos implicated Bicc1 and Dicer functioning together to regulate the *dand5* mRNA (Maerker et al., 2021).

While Bicc1 has primarily been observed to impact mRNAs through mechanisms of repression, in certain contexts it may instead activate the translation of target RNAs. Specifically, in a mechanism involving miRNAs, lower levels of *Pkd2* mRNA and protein were observed In Bicc1−/− mouse embryos (Tran et al., 2010). Follow-up experiments suggested that Bicc1 antagonized *miR-17*’s repressive impact on the *Pkd2* mRNA (Tran et al., 2010). Additional research will be required to elucidate the mechanistic aspects of Bicc1 mRNA activation functions.

## Human BICC1 and clinical associations

Bicc1 was discovered in model organisms in the course of genetic and molecular screens designed to identify biologically important proteins. Subsequently human Bicc1 has been identified in the search for genes responsible for or associated with specific disease states.

### Polycystic kidney disease

Polycystic kidney disease (PKD) is the leading cause of end-stage renal disease in children and adults. The defining characteristics of PKD are an enlargement of the kidneys accompanied by the accumulation of fluid-filled cysts that can be up to several centimeters in diameter (Bergmann et al., 2018). In model organisms mutations of mouse Bicc1 as well as morpholino disruption of *Xenopus* and zebrafish Bicc1 orthologs in developing embryos result in cystic kidneys (Cogswell et al., 2003; Tran et al., 2007, 2010; Bouvrette et al., 2010) that closely resemble the defects due to PKD. Specific BICC1 mutations have been identified in a small number of human patients with cystic kidney dysplasia (Kraus et al., 2012) as well as a cohort of fetuses affected with severe renal defects (Jordan et al., 2022).

### Cholangiocarcinoma

Cholangiocarcinomas are aggressive cancers that form in the bile ducts and account for 3% of all observed gastrointestinal tumors (Schizas et al., 2020; Wang et al., 2021). Patients have a survival rate of less than 10% due to the difficulties in early detection and a lack of effective treatments. One of the most prevalent genome rearrangements that gives rise to cholangiocarcinomas is a fusion between the genes encoding FGFR2 (Fibroblast growth factor receptor 2) and BICC1 that results in the production of an FGFR2-Bicc1 fusion protein (Arai et al., 2014; Ross et al., 2014; Li et al., 2020; Scheiter et al., 2021). The kinase portion of these FGFR2-Bicc1 fusions is constitutively activated due to multimerization activities of the Bicc1 component. As part of the pathology it is the activated kinase that functions as a potent driver of cholangiocarcinoma tumors. Identifying the molecular determinants of Bicc1 multimerization is a potential strategy to develop new treatments for these cancers.

### Major depressive disorder

Major Depressive Disorder (MDD) is a debilitating psychiatric condition that affects millions and significantly reduces their quality of life (Gutiérrez-Rojas et al., 2020; Cai et al., 2021; Abdoli et al., 2022; Shorey et al., 2022). BICC1 gene polymorphisms linked to MDD have been identified in several independent human Genome Wide Association Screens (Lewis et al., 2010; Bermingham et al., 2012; Ryan et al., 2016). Correlative studies of human BICC1 along with experimental investigations in rodent models have revealed potential links between the Bicc1 activity in specific neurons of the brain and severe depression. In humans, elevated levels of *bicc1* mRNA were found in postmortem brain tissues from patients that suffered from severe depression (Ota et al., 2015). In a rat model for depression the experimental reduction of *bicc1* mRNA, including the reduction through treatment with the antidepressant ketamine, significantly diminished depressive behaviors in the animals (Ota et al., 2015). These and other data provide compelling evidence that Bicc1 activity is functionally linked by poorly understood mechanisms to the pathophysiology of MDD.

## Summary and conclusion

Since its discovery in *Drosophila*, Bicaudal-C has subsequently been found in a wide array of different biological contexts. However, in only a small fraction of these has it been established that Bicc1 directly impacts biology by functioning as a post-transcriptional regulator. Some of the most complete information regarding Bicc1 functions has come from the study of *Xenopus* and mouse embryos and the regulated translation of specific mRNAs. In *Xenopus* Bicc1 resides at the center of a post-transcriptional regulatory network present in early stage embryos and this network establishes the proper spatial distributions of cell fate regulatory proteins essential for normal development. The analysis of this network has provided new insights into the regulation of cell fates in vertebrate embryos as well as providing both conceptional and technological foundations to guide the study of Bicc1 in other contexts. In particular, mutations in human BICC1 have been observed in several different disease states, but the functional consequences of these mutations in most cases have been difficult to evaluate. The ability to experimentally analyze particular aspects of Bicc1 functions in model organisms combined with the high degree of evolutionary conservation provides powerful tools to understand how defects in human BICC1 contribute to specific disease states and could lead to new diagnostics and treatments for such disorders.
